# Assessment of Common Factors Associated with Droplet Digital PCR (ddPCR) Quantification of *Paratrichodorus allius* in Soil

**DOI:** 10.3390/ijms25063104

**Published:** 2024-03-07

**Authors:** Bisho Ram Lawaju, Guiping Yan

**Affiliations:** Department of Plant Pathology, North Dakota State University, Fargo, ND 58108, USA; bisho.lawaju@ndsu.edu

**Keywords:** ddPCR, *Paratrichodorus allius*, soil pre-treatment, soil storage, DNA purification, PCR inhibitors, BSA

## Abstract

This research investigated the factors associated with the quantitative detection of *Paratrichodorus allius* in soil using droplet digital PCR (ddPCR). Small-sized nematodes exhibited significantly lower DNA quantities compared to their medium and large counterparts. Soil pre-treatments (room temperature drying and 37 °C oven-drying) demonstrated no substantial impact on ddPCR detection, and soil storage (0–3 months at 4 °C) exhibited negligible alterations in DNA quantities. A commercial DNA purification kit improved the resulting quality of ddPCR, albeit at the cost of a notable reduction in DNA quantity. Upon assessing the impact of inhibitors from soil extracts, a higher inhibitor concentration (5%) influenced ddPCR amplification efficiency. Incorporating bovine serum albumin (BSA) (0.2 μg/μL or 0.4 μg/μL) into the ddPCR setup mitigated the issue. In brief, while ddPCR exhibits minimal sensitivity to soil pre-treatments and storage, higher concentrations of PCR inhibitors and the DNA purification process can influence the results. Despite ddPCR’s capability to detect nematodes of all sizes, quantification may not precisely reflect soil population. Incorporating BSA into the ddPCR setup enhances both detection and quantification capacities. This study represents the first comprehensive investigation of its kind for plant-parasitic nematodes, providing crucial insights for application of ddPCR in nematode diagnosis directly from the soil DNA.

## 1. Introduction

Plant-parasitic nematodes (PPNs) are important soilborne pathogens causing significant crop loss annually by direct damage or facilitating the entry of other pathogens or transmitting viral diseases in the plants. The severity of damage caused by these PPNs depends on nematode population densities, nematode species, host cultivars, and the environment [1]. The traditional methods of nematode extraction, identification, and quantification in soil are based on the active (e.g., Baermann funnel, Whitehead and Hemming method) or passive (sugar-flotation-centrifugation method) collection techniques. Although these are the most frequently used methods of nematode quantification, they have several limitations. These methods require considerable time, labor, and equipment, and the extraction efficiencies vary across labs [2,3]. Moreover, they are based on microscopic counting and identification based on morphological characteristics which can lead to misidentification of the nematodes [2]. To avoid these technical difficulties and misidentification of nematodes, nucleic acid (DNA or RNA)-based molecular techniques have been developed. Nucleic acid sequences provide excellent molecular targets for the detection and identification of nematodes. Polymerase chain reaction (PCR) is the most frequently used molecular technique in pathogen diagnosis [4]. In recent years, more advanced techniques such as real-time PCR [3,5], LAMP [6,7], RPA [8,9], and ddPCR [10,11] are also available that can give qualitative as well as quantitative measurements of the nematodes. These techniques can detect and quantify the nematodes from a pure population, mixed populations, as well as directly from total soil DNA, thus alleviating the need for nematode collection from soil and DNA extraction from nematodes.

DNA extracted from soil for molecular analysis often requires extensive purification due to the intricate nature of soil. Soil comprises various components, including solid-phase inorganic minerals, organic substances like humic acids, and aqueous elements [12], all of which influence DNA retention and extraction efficiency [13]. DNA in soil originates from living organisms such as microorganisms, plants, and animals, existing either within cells or as extracellular DNA bound to soil minerals or freely in solution [14]. Intracellular DNA can be released through physical or enzymatic treatments, while extracellular DNA may bind to soil minerals, hindering enzymatic degradation [15]. Alternatively, unbound DNA can undergo degradation by soil microorganisms or be assimilated into microbial communities. The binding of DNA to soil minerals is influenced by factors like soil type, mineral composition, humic substance concentration, temperature, and pH, impacting DNA extraction efficiency [15,16,17]. Soil’s organic matter, including compounds from living organisms like carbohydrates, lipids, and proteins, also adds challenges for DNA extraction and downstream processing. DNA adsorbed to organic molecules can result in low extraction yields, while water-soluble humic substances can inhibit PCR either directly or indirectly [18]. Despite these challenges, many commercial soil DNA extraction kits are available, capable of yielding satisfactory DNA levels even from challenging samples [13]. Additionally, soil DNA purification kits can effectively remove contaminants, enhancing the purity of extracted DNA [19].

Molecular techniques such as PCR are sensitive and reproducible when analyzing pure DNA samples, but the amplification efficiency and sensitivity are often challenged by PCR inhibitors that come along with the nucleic acids during the extraction process as contaminants [20]. Inhibitors are difficult to completely remove from the sample but certain additives, termed PCR enhancers, can enhance the PCR amplification despite the presence of PCR inhibitors [21]. Several additive substances such as betaine, dithiothreitol (DTT), dimethyl sulfoxide (DMSO), bovine serum albumin (BSA), and glycerol have been used to improve the yield of difficult PCR reactions [22,23,24]. These enhancers facilitate the amplification either by improving the specificity, sensitivity, and efficiency of the reaction, or by neutralizing any inhibitor effects.

In addition to the soil’s intrinsic properties, soil sample collection, storage conditions, and length of storage can also affect the soil DNA analysis. Soil pre-treatments, i.e., soil sample handling, storage, and sample preparation prior to DNA extraction, have been shown to affect soil microbial analysis [25]. Ideally, DNA extraction should be conducted as soon as possible, but when this is not possible, samples should be preserved in an ambient environment. Many PPNs can tolerate periods of desiccation. Previous studies have demonstrated that soil samples intended for nematode analysis can be stored at 5–20 °C for a considerable length of time with the optimum storage temperature being 10–15 °C [26,27]. However, nematode recovery percentage decreases with time regardless of the storage temperature [28]. In most of the studies, the nematode population declined rapidly when stored above 20 °C [29,30]. Although storage at low temperatures was not found to be as destructive as higher temperatures, storing below 4 °C was not recommended [31].

In the studies cited, fresh soil samples were best suited for investigating nematodes in soil [32,33]. Some other studies on soil microbial populations have reported that air-drying did not substantially affect microbial DNA compositions [33,34]. Fresh soil often is more difficult to handle, especially when it contains high moisture. Freezing or refrigerating soil samples needs space and energy. Dry soil is biologically inactive and is therefore easier to handle, store, or transport [25]. Thus, for nematologists or microbiologists, air-drying would be desirable, but further studies may be required to validate air-drying as a routine treatment owing to the diverse nature of samples, organisms, and study objectives.

Droplet digital PCR (ddPCR) is an innovative technique in molecular biology used to precisely measure the absolute quantity of target DNA or RNA molecules in a sample. It overcomes the limitation of traditional PCR methods and provides higher precision and sensitivity compared to other molecular techniques [35]. By partitioning the sample into individual droplets and analyzing them separately, ddPCR achieves accurate quantification even in complex samples [36]. In a short span of time, it has proven to be an invaluable tool in genetic research, clinical diagnosis, environmental monitoring, and food testing [37,38,39,40]. While recent studies have explored ddPCR’s potential in diagnosing PPNs directly from soil [10,11], further investigation is needed to identify the common factors influencing successful ddPCR reactions for detecting and quantifying nematodes.

This study aimed to characterize some common factors that can influence the outcomes of ddPCR reactions for nematode diagnosis directly from soil samples. We used *Paratrichodorus allius*, a stubby root nematode species, as our test plant-parasitic nematode. We evaluated nematode body size/developmental stage, soil pre-treatment, DNA purification, PCR inhibition by soil extracts, and PCR enhancer effects on ddPCR reaction and quantification of *P*. *allius* DNA in total soil DNA. The study provided valuable information on optimal ddPCR reactions with soil DNA samples that would be useful for conducting molecular research with environmental samples.

## 2. Results

### 2.1. Effect of Nematode Body Sizes on Quantitative Detection of Paratrichodorus allius

Individual *P. allius* nematodes were meticulously selected, and their body length and width were measured under a microscope, leading to their categorization into three groups: large (body length > 650 μm), medium (400–650 µm), and small (<400 μm). These nematodes were manually inoculated into nematode-free, autoclaved soil, and subsequently soil DNA was extracted with the DNeasy PowerSoil Pro Kit (Qiagen, Hilden, Germany). The nematode DNA copy number in the soil DNA extracts was scrutinized using ddPCR. Upon analysis, the nematode DNA copy numbers were found to be statistically similar (*p* > 0.05) in large and medium-sized nematodes. However, in contrast, DNA extracts from small-sized nematodes exhibited a significantly lower nematode DNA copy number (Table 1). These findings indicate the subtle relationship between nematode size and DNA abundance.

### 2.2. Soil Pre-Treatment

In this study, naturally nematode-infested field soils from five different locations underwent various treatments, including no-treatment (NT) or fresh soil, air-drying at room temperature (RT), oven-drying (OD), and autoclaving (AC), to analyze the effects of pretreatments associated with soil moisture loss on soil DNA extraction and quantification by ddPCR. Fresh soil with no treatment (NT) was used to compare the results with other treatments. All soil samples had low moisture contents (3.22%) and no significant difference in soil weight loss was observed due to the treatments (*p* > 0.05). Following these treatments, soil DNA extraction was performed, and nematode DNA copy numbers were quantified using ddPCR. The average DNA copy numbers for no-treatment (NT), air-dried at room temperature (RT), oven-dried (OD), and autoclaved (AC) soils were 275.42, 122.99, 208.14, and 1.89, respectively. Statistical analyses at α = 0.05 indicated no significant differences in DNA copy numbers among NT, RT, and OD soils. However, the autoclaved (AC) treatment resulted in a significant reduction in DNA copy numbers in the soil compared to the no-treatment (NT) soil (Table 2). These findings suggest that while the overall moisture levels remained low, only autoclaving had a substantial impact on nematode DNA abundance in the soil.

### 2.3. Effect of Soil Storage Time

**Figure 1:**
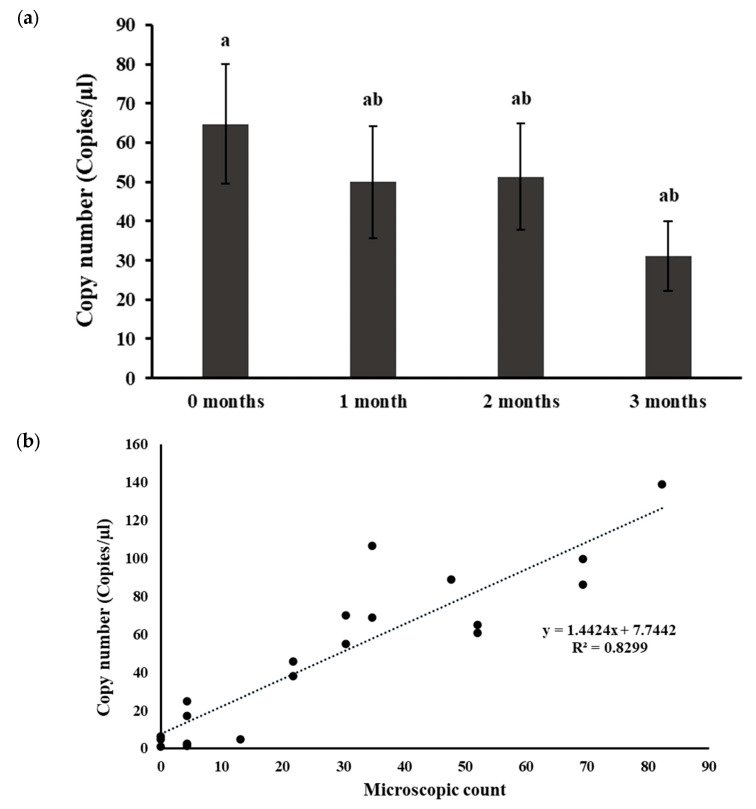
Effect of soil storage time in the quantification of *Paratrichodorus allius* DNA in soil DNA extracts. (**a**) Nematode DNA copy numbers determined by ddPCR in soils stored at varying time. Different letters above the bars indicate statistically significant difference according to Tukey-Kramer grouping for least square (LS) means. Bars are the mean ± standard error of N = 15. (**b**) Correlation of the DNA copy numbers quantified by ddPCR with traditional nematode extraction and microscopic counts. Naturally nematode-infested field soil samples were stored at 4 °C for varying time. At each time point, nematodes were extracted from the soil using the sieving and sugar -flotation-centrifugation method, followed by microscopic counts. Simultaneously, soil DNA was extracted at each time point using the Dneasy PowerSoil Pro Kit, and ddPCR reactions were conducted to quantify nematode DNA copy numbers in the soil DNA extracts. The dotted line represents the line of best fit from the correlation analysis between the microscopic counts and the associated nematode DNA copy numbers quantified by ddPCR.

In this study, five distinct field soil samples were stored at 4 °C for 0–3 months. Soil DNA extraction was performed at each time point, and nematode DNA was quantified using ddPCR. While a gradual decrease in nematode DNA within the soils was observed over the storage period (Figure 1a), statistically, the differences were not significant (*p* > 0.05). Additionally, nematodes were extracted from the soils using the sieving-sugar-flotation-centrifugation method at the respective storage times, and significantly lower nematode numbers were observed at 2 and 3 months compared to the counts at 0 months (Supplementary Table S1). These nematode count data were employed to establish the correlation between the DNA copy numbers obtained from ddPCR and the nematode numbers determined through manual extraction and microscopic counting (Figure 1b). A strong positive correlation (*R*^2^ = 0.83) between the DNA copy numbers quantified from ddPCR and the traditional microscopic counting, as described by y = 1.4424x + 7.7442, was evident (Figure 1b). This observation underscores the reliability of ddPCR as a method for quantifying nematode DNA, and although storing the soil samples at 4 °C gradually reduced the quantifiable nematode DNA over time, it may not significantly affect nematode DNA copy number estimation by ddPCR.

### 2.4. Effect of Soil DNA Purification

**Figure 2:**
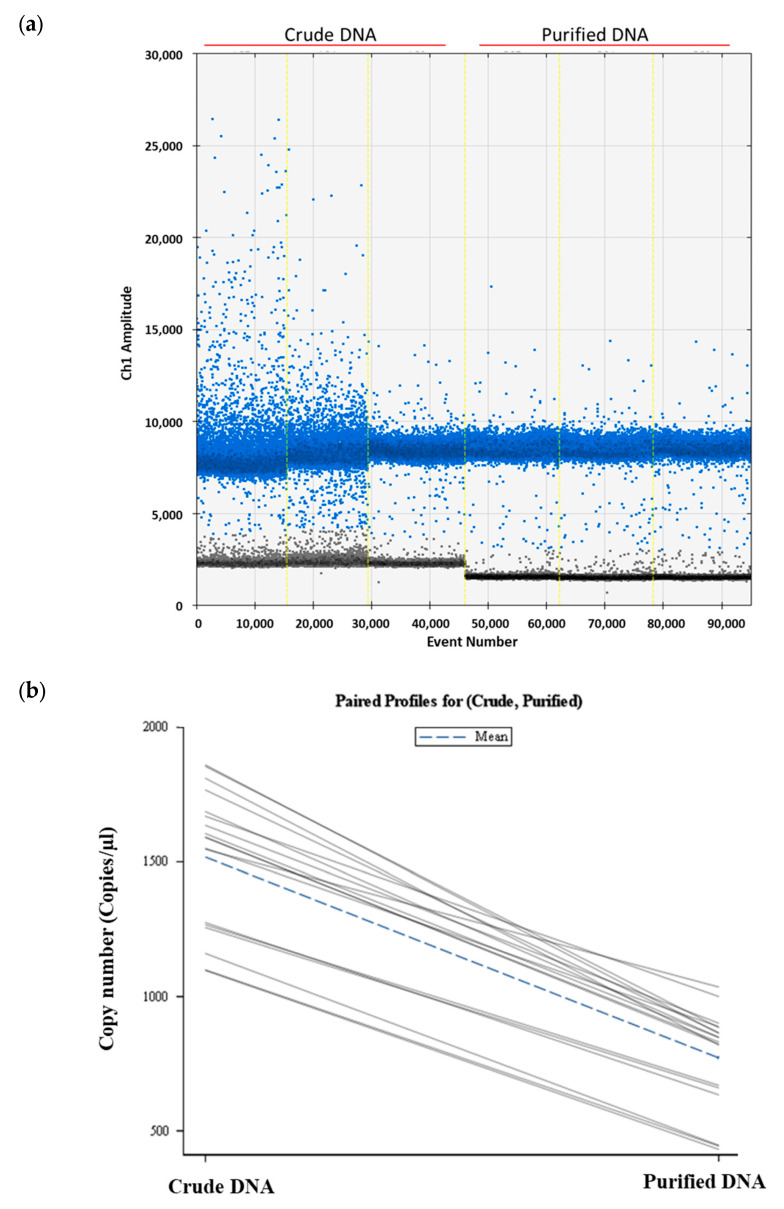
Effect of DNA purification process in the quantification of *Paratrichodorus allius* DNA in soil DNA extracts. (**a**) One-dimensional amplitude plot obtained from ddPCR reactions showing variations in scatteredness and amplitudes of positive droplets (blue dots) in crude DNA and purified DNA. (**b**) A paired profile plot showing individual changes in *P. allius* DNA copy numbers due to soil DNA purification. Soil DNA was extracted from artificially nematode-inoculated soil (five or ten nematodes per sample) using the Dneasy PowerSoil Pro Kit (Qiagen). The resulting soil DNA extract was considered crude DNA. Crude DNA was further cleaned up with the Dneasy PowerClean Pro Cleanup Kit (Qiagen) to obtain the purified DNA. Both crude and purified DNA were analyzed with ddPCR for quantitation of nematode DNA copy numbers. In the above graph (**a**), amplitudes of positive droplets (blue dots) and negative droplets (grey dots), generated in ddPCR by three biological DNA samples from 10 nematodes in crude form and purified form are presented. Event number is the number of droplets read in total for each lane in ddPCR and the vertical light-yellow lines separate different samples. In the paired profile plot (**b**), each line represents a mean value of a paired observations across different conditions and the plot provided the visual representation of how DNA copy numbers varied with crude (**left**) and purified DNA (**right**).

Total soil DNA was extracted from soils manually inoculated with five and ten *P. allius* nematodes in three replicates using the Dneasy PowerSoil Pro Kit. Half of the extracted soil DNA was utilized in ddPCR as crude DNA, while the remaining portion underwent purification with the Dneasy PowerClean Cleanup Kit prior to ddPCR analysis. The ddPCR assay exhibited higher fluorescence signals and amplitudes with crude DNA extracts compared to purified DNA (Figure 2a). Although clear gaps between the positive and negative droplets were evident in both crude and purified DNA samples, there were more scattered positive droplets (blue dots) with crude DNA samples. The fluorescence amplitudes of negative droplets (grey dots) in crude DNA were relatively higher compared to the negative droplets in purified DNA samples. Numerically higher DNA copy numbers were calculated in soil DNA extracts harboring ten individuals of *P. allius* (1598.04 ± 261.59 copies/μL), but statistically they were similar to soil DNA extracts with five individual *P. allius* (1437 ± 243.06 copies/μL). Similar to the crude soil DNA extracts, the DNA copy numbers determined by ddPCR in purified DNA were not different between the two sets of nematode numbers (787.87 ± 101.99 vs. 755.21 ± 246.19). However, when comparing crude and purified DNA samples, a significant reduction (*p* < 0.05) in target DNA copy numbers was observed with purified DNA samples (Figure 2b).

### 2.5. Effect of PCR Inhibitors Co-Extracted along with Soil DNA

**Figure 3:**
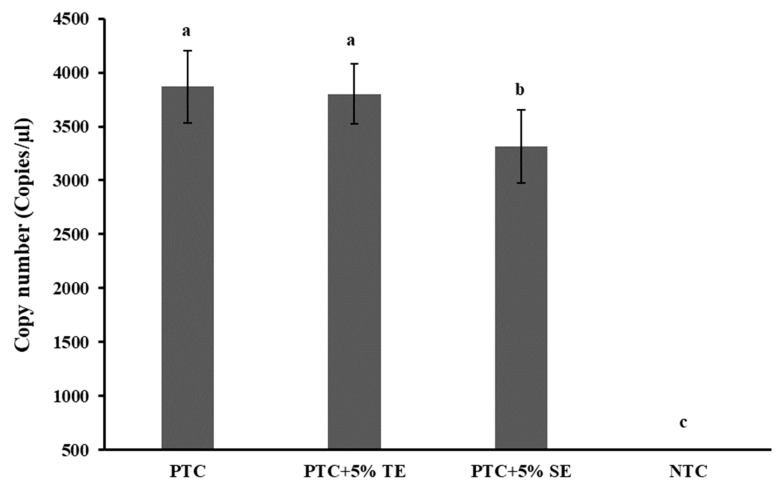
Effect of PCR inhibitors from soil extract on *Paratrichodorus allius* DNA quantification by ddPCR. Positive template control (PTC) contained only the amplicon derivatives (obtained with the *Paratrichodorus allius*-specific primers PaF11/PaR12, products confirmed by gel electrophoresis, and further validated by sequencing), without TE or SE. Soil extract (SE) was prepared from nematode-free field soil with 1X Tris EDTA (TE) buffer (pH 8.0) in a 1:1 (*w*/*v*) ratio. A concentration of 5%SE with PTC was obtained by adding 1 µL of stock SE to the 20 µL reaction volume for ddPCR. A concentration of 5%TE with PTC was obtained like the 5%SE in PTC and was included in the study to investigate the potential inhibitory effect of TE alone on ddPCR. NTC was the no-template control in which nuclease-free double-distilled water was added as template DNA. Different letters above the bars indicate statistically significant difference according to Tukey-Kramer grouping for least square (LS) means. Bars are the mean ± standard error of N = 9.

Field soil from Sargent County, ND was used for soil extract preparation. The soil was loamy sand (86–96% sand, 5–11% silt, 3–5% clay, 0.9–1.0% organic matter, 0.38–0.67% humic acids, pH 5.5–5.7) [27] planted with corn and potato in rotation. Before subjecting it to the extract preparation with Tris EDTA (TE) buffer, the soil was confirmed to be devoid of *P. allius* nematodes by traditional nematode extraction and microscopic observation methods. The soil extract, thus prepared, underwent ddPCR analysis to evaluate its inhibitory effects, using a positive template control (PTC) sample. Varied volumes of soil extract were introduced into the ddPCR reaction mixture, maintaining a consistent final reaction volume of 20 μL with diverse concentrations of the soil extract. Among the examined concentrations (1–5%) (Supplementary Figure S1), only the 5% soil extraction concentration demonstrated a significant reduction (*p* < 0.05) in DNA copy numbers in ddPCR compared to the positive template control without soil extracts (0% soil extract), as illustrated in Figure 3. Additionally, a positive sample with 5%TE buffer was included to assess any potential impact of TE buffer alone on target DNA quantification in ddPCR. Encouragingly, no significant effect (*p* > 0.05) was observed when incorporating 5%TE buffer into the reaction, affirming its minimal influence on the ddPCR analysis.

### 2.6. Effect of Bovine Serum Albumin (BSA)

**Figure 4:**
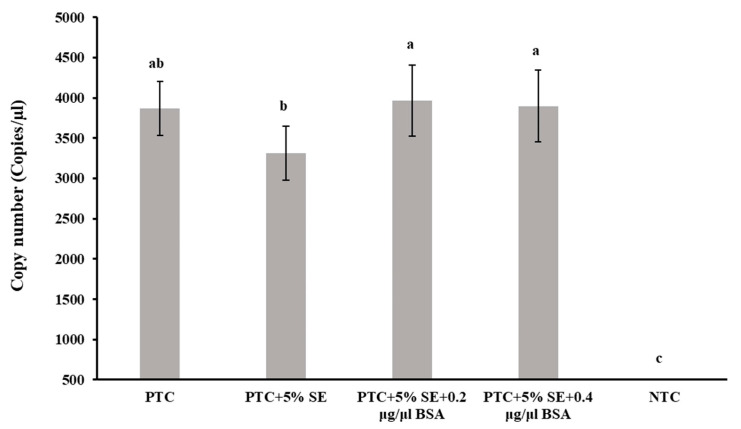
Effect of BSA on *Paratrichodorus allius* DNA quantification by ddPCR in the presence of inhibitors from soil extract. Positive template control (PTC) contained only the amplicon derivatives (obtained with the *Paratrichodorus allius*-specific primers PaF11/PaR12, products confirmed by gel electrophoresis, and further validated by sequencing), but no other additives such as SE or BSA. Soil extract (SE) was prepared from nematode-free field soil with 1X Tris EDTA (TE) buffer (pH 8.0) in a 1:1 (*w*/*v*) ratio. A concentration of 5%SE with PTC was achieved by adding 1 µL of stock SE to the 20 µL reaction volume for ddPCR. Bovine serum albumin (BSA) concentrations of 0.2 µg/µL and 0.4 µg/µL in ddPCR were achieved by incorporating 0.4 µL and 0.8 µL of stock solution (10 µg/µL) in 20 µL ddPCR reaction volume, respectively. Different letters above the bars indicate statistically significant difference according to Tukey-Kramer grouping for least square (LS) means. Bars are the mean ± standard error of N = 9.

A significant reduction (*p* < 0.05) in quantifiable DNA copy numbers in the positive template control was observed in the presence of 5% of the soil extract, prompting the addition of BSA to investigate its potential to neutralize the inhibitory effects of the soil extract. Two concentrations of BSA (0.2 μg/µL and 0.4 μg/μL, respectively) were introduced into the ddPCR reaction mixtures, and the resulting DNA copy numbers were analyzed. The results demonstrated that the inclusion of both concentrations of BSA in the ddPCR reactions with 5% soil extract led to comparable levels of nematode DNA as observed in reactions with positive template control without 5% soil extract (Figure 4).

## 3. Discussion

An earlier study has shown that nematode body size was influenced by available food, sex, and growth temperature. However, there was no clear correlation between the body size and the genetic relationships or genome size of the nematodes [41]. Even with the two nematode species, *Pratylenchus neglectus* and *P. penetrans,* very similar quantities of DNA were detected in adult females, second-stage juveniles, and eggs, suggesting no variations in nucleic acid quantities based on different life stages of a nematode [2,3]. However, another study reported a significantly higher amount of *P. penetrans* DNA in a male and a female nematode compared to a small juvenile [42]. Consistent with the result, *P. allius* has also been reported to contain a significantly low amount of DNA in small-sized juveniles compared to larger juveniles or intermediate/larger females [32]. In all these studies, DNA was measured by qPCR. In our study, *P. allius* DNA from different body sizes was measured for the first time using ddPCR, an advanced technique compared to qPCR, and the results were in coherence with previous findings. Similar quantities of DNA were measured in large- and medium-sized nematodes, but smaller DNA amounts were found in small-sized nematodes. These results suggested that the total nematode DNA yield from soil may vary based on nematode developmental stage as well as nematode species and the soil matrix. In nematodes, as in many organisms, cell division and growth contribute to overall body size. Thus, larger-sized nematodes may possess more cells, leading to a higher total DNA content [43]. Also, during development, nematodes undergo significant changes in cell proliferation, differentiation, and growth. The growth of gonads, developing embryos, and egg sacs in the adult nematodes may be attributed to the increased DNA contents in adult nematodes compared to the young juveniles [32,44]. Hence, in an environment containing various stages of nematodes, the nematodes could be detected by ddPCR, but the actual number of nematodes from the traditional counting may differ from the number estimated by ddPCR.

In our investigation, the moisture content across all soil samples consistently remained low (~3%), and none of the pre-treatments resulted in any noteworthy variance in moisture loss. Likewise, both air-drying at room temperature (25 °C) and oven-drying (37 °C) showed no significant impact on the quantification of *P. allius* in soil DNA extracts through ddPCR. Numerically, fresh soil without any treatments yielded higher nematode DNA copies in ddPCR, followed by oven-dried soil and air-dried soil. Autoclaved soil served as a negative control, yielding the lowest nematode DNA quantities, as expected. These findings align with previous studies utilizing DNA-based methods for analyzing soil microbial communities, which have indicated that air-dried soil can be stored at room temperature for an extended period without altering microbial community structures [33,34,45]. Similarly, studies focused on PPNs have demonstrated that both air-dried soil [42] and oven-dried soil [2] do not yield significant differences in nematode quantification when employing quantitative PCR (qPCR). Researchers attribute the enhanced detection capacity to the ease of handling and processing dried samples, reduced microbial activities, and the uniform dispersal of organisms within homogenized dried soils. Our results indicate that fresh soils in their natural conditions can be used to detect and quantify nematodes by ddPCR. However, recognizing the challenges associated with on-site analyses and sample handling, such as high moisture and difficulties in sieving the soils, our study suggests that reducing soil moisture could be a practical strategy to alleviate difficulties in shipping or handling samples without considerable effects on DNA quantification. However, to generalize this notion, additional studies would be necessary. The soils analyzed in this study were all the same type with lower moisture contents than usual. The relatively lower moisture level might have induced dormancy in the nematode population, thereby not exhibiting significant differences in DNA copy numbers among fresh soils, air-drying at room temperature, and oven-drying at 37 °C.

Temperature is important for the survival, growth, and development of all organisms, including nematodes, but they may respond to temperature differently even within the same genus [30]. Entomopathogenic nematodes were found to survive well in lower temperatures (5 °C) compared to higher temperatures (35 °C), but their pathogenicity was greatly reduced [29]. In studies of storage temperature effects on PPNs, the optimal storage temperature was found to be 10–15 °C [27,28,30,31]. Nematodes were rapidly killed in freezing temperatures [27] and above 20 °C [30]. Interestingly, *Pratylenchus* species, one of the common PPNs, were found to be infective even after 36 months of storage at 12.5 °C [26]. Whether nematodes are dead or alive in stored soil, the molecular analysis of nematodes in soil should not be affected substantially by the physical conditions of the nematodes since DNA from both dead and live nematodes would be accounted for in the analysis. However, increased temperature accompanied by humidity may increase microbial activities, thus increasing DNase activity and reducing the half-lifetime of DNA, which may lower the extractable DNA from the soil affecting molecular analysis [15].

In addition to storage temperature, storage time also affects the molecular analysis of the soils. Several reports are available on storage time regarding microbial community analysis and nematode community analysis. Although short-term storage of soils at −20 °C, 4 °C, lyophilized, and at room temperature did not change the quality of DNA extracts for molecular analysis [33], long-term storage affected detectable community structures [45]. In contrast, microbial community composition was achieved from archived soils (dried and stored for more than 50 years) [34]. Thus, the storage time may not directly influence the molecular analysis of soil, but other environmental factors may, such as soil pH, soil type, vegetation, and soil handling before storage [45]. In all studies with PPNs, nematode numbers gradually decreased over time even at the optimal storage temperature [26,27,28,31]. In a proteomic study with entomogenic nematodes at two storage temperatures (9 °C and 20 °C), it was found that the proteome of the nematodes was affected by temperature, as well as nematode species [46]. In our study, soil was stored at 4 °C for three months. *P. allius* and their DNA copy numbers were quantified every month by manual extraction and microscopic count and by ddPCR, respectively. Manual extraction and microscopic counting showed a significant decline in nematode counts over time, while ddPCR quantification showed a gradual decrease without significant differences over three months (Supplementary Table S1). The likely reason for insignificant lowering of counts over three months estimated by ddPCR could be the accounting of nematode DNA from all possible forms (alive, dead, eggs, or deformed) by ddPCR in contrast to the microscopic count of only the identifiable juveniles and adult nematodes. Moreover, nematode DNA copy numbers quantified by ddPCR were in good correlation with the microscopic count (*R*^2^ = 0.8299) and suggested soils could be stored for at least three months in a refrigerator for molecular analysis. Again, it should be noted that the ddPCR quantification across storage time may vary for soil types with different moisture levels at the time of sampling. Although five different soil samples were analyzed in the study, they were all the same soil type and had similar moisture levels. The relatively lower moisture level might have already induced stress and dormancy in the nematode population, leading to reduced microbial and enzymatic activities, which could explain the relatively insignificant differences in DNA copy numbers over time at 4 °C. To confirm that storage time would not have an effect on quantifiable nematode DNA by ddPCR, further studies with varied soil types, moisture levels, and different levels of nematode populations may be required.

Acknowledging the intricate nature of soil samples, DNA purification becomes imperative due to the potential coextraction of substances such as humic acids, polysaccharides, urea, phenolic compounds, and heavy metals. Failure to remove these compounds can result in PCR inhibition [47]. Therefore, the importance of purification in eliminating inhibitory substances from soil DNA samples is underscored [13]. Rapid DNA purification kits are available these days, but a significant portion of DNA is usually lost and DNA degradation or damage during the process is quite common during the process [48]. In our study as well, a decrease in DNA copy numbers was observed in purified samples compared to their respective crude samples, indicating a loss of DNA during the purification steps (Figure 2b). However, in the amplitude graph (Figure 2a), compact positive droplets (blue droplets) were observed with purified DNA samples. The gap between baseline (grey negative droplets) and positive droplets was increased and rains (droplets with intermediate amplitudes between positive and negative droplets) were decreased by purification. We used a SYBR Green-based product, EvaGreen Supermix, for DNA quantification in ddPCR that can bind to any double-stranded DNA. The possible reasons for the higher copy number detection in crude DNA might be higher concentrations of target DNA as well as the chemistry of the SYBR Green. Crude DNA extract could contain target DNA plus non-target DNA, genomic DNA, and degraded DNA which cannot be differentiated by SYBR Green, resulting in increased positive droplets. Additionally, these components, along with residual contaminants and PCR inhibitors present in crude DNA, can lead to non-specific amplification or interference with the amplification reaction, resulting in increased background fluorescence levels in negative droplets, leading to elevated baseline amplitudes (as exhibited in Figure 2a). During purification, some amount of target might be lost, but many other impurities are also removed, thus eliminating the unspecific bindings of the dye to the impurities and reducing the number of tentative positive droplets. Due to the availability of many commercial kits standardized for soil DNA extraction and their improved protocols, soil DNA purification may not be required for the general diagnosis of nematodes from soil samples. These kits feature column-based or silica gel-based purification steps that remove considerable amounts of inhibitors from the extracts. In cases where the sample has a higher amount of impurities resulting in amplification failure, or if an in-house manual extraction protocol is followed, additional purification may be necessary. Nonetheless, it is important to acknowledge that purification comes at the cost of a reduction in target concentrations.

Humic substances (HS) are one of the most common inhibitors existing in environmental soils [49]. They can be humic acid (HA), fulvic acid (FA), or humin [50]. Humin is an insoluble substance and thus does not affect PCR amplification, but both HA and FA are dibasic weak acids, which can coprecipitate with DNA influencing the PCR reactions [51]. In this study, soil extract prepared by mixing nematode-free field soil with Tris EDTA (TE) buffer was used for evaluating the performance of soil inhibitors in ddPCR. TE buffer (pH 8.0) was used for this purpose since humic acids are soluble in neutral and alkaline pH, whereas fulvic acid is soluble in all pH [50,52]. Moreover, TE is one of the common buffers used for DNA elution and long-term storage of DNA. Soil extracts in different concentrations (1–5% to final reaction volume) compared to a positive template control were run in ddPCR (Supplementary Figure S1) and the incorporation of 5% of soil extract significantly lowered the copy number detection compared to the sample without soil extract. TE buffer at 5% (50 µM of EDTA to the final reaction volume) was also run with a positive template control to assess if the concentration of EDTA used to prepare soil extract could itself inhibit the amplification. EDTA is known to inhibit PCR for a long time at a concentration above 1 mM [49]. In our study, the addition of 5% TE buffer alone did not influence ddPCR reactions but 5% of soil extract prepared with TE buffer significantly lowered the DNA copy number detection in ddPCR. This reduction could be attributed to the inhibitors extracted from the soil with TE buffer, most probably the humic acids and fulvic acids. ddPCR is considered tolerant to many inhibitors [35,36,53] as the inhibitors are also partitioned into many individual PCR chambers limiting the inhibition effect without affecting the end-point detection. Thus, inhibitor levels that affect amplification in normal PCR or real-time PCR may not affect ddPCR, but excess levels of inhibitors would affect ddPCR as observed in our study with 5% of soil extract.

Different additives are known to improve the yield of PCR products derived from complex materials. Several studies have reported the PCR-enhancing nature of bovine serum albumin (BSA) for difficult samples [22,23,24]. BSA is considered to stabilize polymerase, and inactivate phenolic compounds (tannic acid, humic acid, fulvic acid) and endogenous proteases [21,49], but the concentration of BSA to optimize amplification varied among studies. BSA is considered to have a neutral effect on PCR in the absence of inhibitory substances in the reaction. Because it is heat-labile, it can enhance amplification only in the initial cycles of the PCR; thus, running more cycles with BSA may not improve the result [22]. Although BSA enhances PCR reactions in the presence of PCR-inhibiting substances, BSA concentrations above 25 μg/μL can inhibit the reaction [54]. Usually, a concentration of BSA ranging from 0.1–0.4 μg/μL in PCR can relieve inhibition from inhibitors in environmental samples [24], with the optimum being 0.2–0.4 μg/μL to alleviate inhibition from humic acids [49]. In this study, we analyzed two different concentrations (0.2 µg/μL and 0.4 µg/µL) of BSA based on the literature for amplifying environmental samples [24,49]. BSA was incorporated into ddPCR reactions along with 5% soil extracts. Initially, the addition of 5% soil extract had significantly lowered the DNA copy numbers compared to the sample without soil extract but the incorporation of either concentration of BSA in the sample with 5% soil extract reinstated the DNA copy numbers at levels similar to the positive template control (PTC) without soil extract (SE). The differential effects of 0.2 µg/µL and 0.4 µg/µL were not visible in DNA copy numbers, but in the fluorescence amplitude graph (Supplementary Figure S2) the fluorescence amplitude was lowest in the sample with 5% soil extract followed by 5% soil extract + 0.4 µg/µL BSA and 5% soil extract + 0.2 µg/µL BSA. The result suggested 0.2 µg/µL BSA was best for the sample and a higher concentration of BSA can affect the quality of the result. Although 0.2 µg/μL was found optimum for this study, for other samples, it may be necessary to optimize the concentration. Soil type, soil DNA extraction methods, and sample preparation steps can result in variation in DNA quantity as well as PCR inhibitor amounts in the extracts. Thus, to identify the optimum concentration of BSA for enhanced PCR, a series of PCR tests with varying concentrations of BSA may need to be run before finalizing the optimum BSA concentration.

In summary, certain common factors associated with *P. allius* quantification in soil by the ddPCR method were studied. Nematode body size affected the nematode quantification by ddPCR. Soils could be air-dried or oven-dried for DNA-based nematode quantification. In case of delayed processing, the soil could be stored at 4 °C for at least three months without greatly affecting the nematode quantification. Soil DNA obtained from a commercial kit usually does not require purification as ddPCR is less affected by contaminants and soil inhibitors, but higher concentrations of inhibitors may affect the result. The addition of BSA is recommended when soil inhibitors are present. The findings would be useful for future studies to set up and optimize ddPCR methods for soil samples.

## 4. Materials and Methods

### 4.1. Soil Sample

Soil samples were collected in August 2022 from a potato field in Sargent County, ND, where *P. allius* was initially reported in 2016 [55]. At five different sampling plots, the topsoil layer (~2 cm) and associated plant debris were removed, and around 10 kg of soil was collected from each plot and placed in individual plastic sampling bags. The soil samples in the air-tight plastic bags were labelled with information detailing the sampling location and date, and subsequently transported to the laboratory in a cooler for preservation. The soil samples were then stored at 4 °C until further processing.

### 4.2. Nematode Extraction, Identification, and Microscopic Count

The soil sample in each plastic bag was mixed thoroughly and triplicates of 200 g of soil were taken for nematode extraction. The extraction process involved sieving the soil through a stack of sieves, starting with No. 20 (sieve opening 850 µm) above No. 635 (sieve opening 20 μm). The nematode suspensions collected in the No. 635 sieve underwent purification through the sugar-centrifugal-flotation method [56]. Stubby root nematodes (*P. allius*) were morphologically identified and quantified under a dissecting microscope (Zeiss Stemi 305 Lab Microscope; Zeiss, Thornwood, NY, USA). For species identification, DNA from individual nematode was extracted using the proteinase K method [57]. A polymerase chain reaction (PCR) was then conducted with *P. allius*-specific primers PaF1: 5′-AAGCTTGCTGGTAGTTTGTTGG-3′ and PaR12: 5′-AAGTAGTTAAAAGGGGAGTCG-3′, targeting the 247 bp of ITS1 rDNA region of the nematodes [5]. Successful PCR reactions were confirmed by gel electrophoresis and further validated by sequencing the PCR products. The sequenced results underwent a BLAST search with NCBI GenBank-deposited sequences to confirm the species. Confirmed *P. allius* species-specific PCR products were purified, diluted to an appropriate concentration, and utilized as positive controls (hereafter referred to as positive template controls) in subsequent reactions.

### 4.3. Soil DNA Extraction for Nematode Quantification by ddPCR

The DNeasy Power Soil Pro Kit (Qiagen, Hilden, Germany) was employed to extract DNA from soil with a slight modification to the manufacturer’s instructions. Unlike the recommended 0.25 g of soil, 0.5 g of soil was utilized, and at the last step of the DNA extraction procedure, DNA was eluted in 50 µL nuclease-free water. For naturally infested soil, 0.5 g of air-dried, well-ground soil was properly subsampled and subjected to DNA extraction. In case of artificially inoculated soil, a varying number of nematodes were manually introduced to 0.5 g of soil (previously confirmed to be devoid of stubby root nematodes and sterilized by autoclaving twice at 121 °C for 20 min before inoculation) in a PowerBead Pro tube for DNA extraction. The FastPrep-24 5G Bead Beating Lysis system (MP Biomedicals, Santa Ana, CA, USA) with a program set as three cycles of homogenization at the speed of 6 m/s for 40 s with a pause time of 45 s between each cycle was used to homogenize the soil samples. DNA was extracted from three biological replicates for each sample and the isolated DNA was stored at −20 °C until further use.

### 4.4. ddPCR Assay Condition and Process

*Paratrichodorus allius*-specific primers PaF1/PaR12 [5] were employed in the ddPCR assay for nematode quantification. The assay was conducted in a QX200 Droplet Digital PCR system (Bio-Rad Laboratories, Hercules, CA, USA) as described elsewhere [11]. The ddPCR reaction mixture comprised 10 μL of QX200 ddPCR EvaGreen Supermix, 1 µL (100 nM) of each forward and reverse primer, 1 μL of DNA sample, and 7 µL of nuclease-free water, resulting in a final volume of 20 μL. This mixture, along with 70 µL of droplet generation oil in a DG8 cartridge (Bio-Rad), was used to generate droplets in a Bio-Rad automated QX200 Droplet Generator according to the manufacturer’s protocol. The resulting emulsions of microdroplets (40 μL) were transferred to a 96-well PCR plate, which was heat-sealed with a pierceable foil in a PX1 PCR Plate Sealer (Bio-Rad) at 85 °C for 5 s.

The PCR plate was subsequently placed in a Bio-Rad C1000 Touch Thermal Cycler and amplified under the following conditions: 95 °C for 5 min, 40 cycles of 95 °C for 30 s, 54.3 °C for 1 min, followed by signal stabilization at 4 °C for 5 min, 90 °C for 5 min, and hold at 12 °C, with a ramp rate of 2 °C/s in each step. After amplification, the droplets in each well were read using a QX200 Droplet Reader (Bio-Rad) and the target copy numbers were calculated using QuantaSoft software Version 1.7.4 (Bio-Rad).

### 4.5. Evaluation of Various Factors on ddPCR

#### 4.5.1. Nematode Body Size

*Paratrichodorus allius* were classified into three size categories based on body length: large (body length > 650 µm), medium (400–650 μm), and small (<400 μm). This categorization was undertaken to examine statistical differences in quantitative detection using the ddPCR assay. Each individual *P. allius* was hand-picked under a light dissecting microscope (Zeiss Stemi 305 Lab Microscope) and the body length was measured under a Zeiss Axio Scope A1 microscope using ZEN 3.0 (blue edition) image acquisition, processing, and analysis software.

Subsequently, a single nematode from each category was collected and inoculated into 0.5 g of autoclaved field soil (soil devoid of stubby root nematodes and sterilized by autoclaving twice at 121 °C for 20 min before inoculation). The DNA extraction from the soil was carried out using the DNeasy PowerSoil Pro Kit (Qiagen). A total of 30 biological replicates, with 10 from each size category, had their DNA extracted, and the ddPCR assay was executed with three technical replicates. This approach aims to discern potential variations in detection efficiency across different size categories of the nematodes.

#### 4.5.2. Soil Pre-Treatment

The soil sample collected from the field underwent three distinct treatments before extracting soil DNA for nematode quantification by ddPCR. Initially, the soil in the original collection bag was thoroughly mixed and then evenly distributed into three glass petri dishes, each containing 25 g of soil, for three different soil treatments: air-drying at room temperature (25 °C) for 24 h (RT), oven-drying at 37 °C for 24 h (OD), and autoclaving at 121 °C for 20 min (AC). Subsequent to each treatment, the soil weight was measured to ascertain soil moisture levels, and the soil was ground to a fine texture using a porcelain mortar and pestle. Soil DNA was then extracted in triplicates from the finely textured soil, following the previously described method. Additionally, soil DNA from fresh soil (no-treatment, NT) was employed to facilitate a comparative analysis of the results obtained from the different treatments. For this study, five different field soil samples underwent various treatments. After the treatments, soil DNA was extracted in triplicates, and ddPCR was performed in a single replicate from each DNA sample. This comprehensive approach ensures a thorough examination of the effects of distinct treatments on soil samples and a foundation for subsequent analyses.

#### 4.5.3. Soil Storage Durations

The field soil samples from five different locations were stored in separate plastic sampling bags at 4 °C for various durations, including 0 months (fresh soil), 1 month, 2 months, and 3 months. At each designated time point, 25 g of soil from the stored bags was properly subsampled and allowed to air-dry at room temperature for 24 h. The following day, the soils were finely ground, and soil DNA was extracted in triplicates for nematode estimation by ddPCR. ddPCR was run in a single replicate from each DNA sample. Additionally, nematodes were extracted from 200 g of soil in triplicate at each time point using the sugar-centrifugal-flotation method. This extraction allowed for a comparison of microscopic counts with ddPCR results at varying lengths of storage. This systemic approach ensures an examination of the impact of storage on nematode populations.

#### 4.5.4. Soil DNA Purification

The negative control soil without *P. allius* infestation was first confirmed by repeated extraction of nematodes from 200 g of soil by sugar-centrifugal-flotation and microscopic observation. No observation of a single stubby root nematode in all three extracts was considered as a negative control soil. The negative control soil was autoclaved twice at 121 °C for 20 min and was used for manual inoculation of *P. allius* nematode and successive soil DNA extraction process. The stubby root nematodes (5 or 10 in number) were manually inoculated into 0.5 g of autoclaved negative control soil in a PowerBead Pro tube (Qiagen) and soil DNA was extracted using the DNeasy PowerSoil Pro Kit (Qiagen). At the ultimate step of DNA extraction process, DNA was eluted with 100 µL of nuclease-free water, of which 50 µL was used as crude DNA extract, whereas remaining 50 μL was used for DNA purification process with the DNeasy PowerClean Pro Cleanup Kit (Qiagen). Pure DNA was also eluted to 50 μL with nuclease-free water. Both crude DNA and purified DNA were run in three replicates in ddPCR assay. For the analysis, soil DNA was extracted in triplicate from manually inoculated soil (three DNA samples each from soils inoculated with five and ten nematodes), and the ddPCR reactions were performed in three technical replicates.

#### 4.5.5. PCR Inhibitors from Soil Extract

Soil extract was prepared with some modifications to the method described elsewhere [58]. Field soil from Sargent County, ND, confirmed to be without *P. allius* nematodes through conventional nematode extraction and microscopic observation, was mixed with 1X Tris EDTA (TE) buffer in a 1:1 (*w*/*v*) ratio. The mixture was stirred at 225 rpm for 2 h at room temperature in a Thermo Scientific Cimarec Stirring hotplate. Subsequently, the suspension underwent centrifugation at 10,000× *g* for 10 min at 4 °C. The resulting supernatant was collected and filter-sterilized through a 0.2 µm pore-sized sterile syringe filter (VWR). The sterilized supernatant was then stored at −20 °C until used.

For ddPCR reactions, positive template controls served as the template. The template DNA, along with varying amounts of soil extracts (1%, 2%, 3%, 4%, and 5%), were added to the reaction mixture of 20 μL for ddPCR. Each concentration was run in triplicate to investigate the impact of soil extracts on the amplification of the targets.

#### 4.5.6. Bovine Serum Albumin (BSA), a PCR Enhancer

The potential mitigating effect of BSA on PCR inhibitors present in soil during ddPCR assays was investigated. In this context, the positive template control served as the template DNA, while a 5% soil extract functioned as the PCR inhibitor in the ddPCR reactions. Two concentrations of BSA were introduced into the ddPCR reactions. The ultrapure BSA solution (50 mg/mL; Invitrogen, Carlsbad, CA, USA) was diluted 1:5 with nuclease-free water, resulting in a stock solution of 10 μg/μL. This stock solution was then integrated into the 20 μL ddPCR reaction mix to make final concentrations of 0.2 μg/μL and 0.4 μg/μL. Subsequent ddPCR reactions were conducted in triplicate for each concentration, following the previously outlined protocol. The primary objective of the investigation was to clarify the impact of BSA on mitigating PCR inhibitors in soil extracts, ultimately enhancing the accuracy and reliability of ddPCR results.

### 4.6. Statistical Analysis

All the data were analyzed using statistical analysis software, SAS 9.4 (SAS Institute Inc., Cary, NC, USA) using the PROC GLIMMIX procedure to assess the potential statistical differences between the treatments. Post hoc analyses were performed to compare least square means (LS-means) among the treatment groups, and significance differences were determined using the Tukey–Kramer method at a 95% confidence level. For comparison of means from related groups (e.g., with and without DNA purification), a paired *t* test was performed, and *p* value was determined to check the significance of the differences.

## Figures and Tables

**Table 1:** DNA copy numbers from DNA extracts of soil inoculated with single *Paratrichodorus allius* nematodes with different body sizes using ddPCR.

Category ^Z^	Length (µm) ^y^	Width (µm) ^y^	Mean Copy (DNA Copies/µL ± SD) ^x^
Large	658.02–827.45	31.35–43.51	38.76 ± 22.53 a
Medium	438.72–623.01	26.67–38.21	39.21 ± 20.68 a
Small	324.22–397.09	19.63–34.92	8.54 ± 6.01 b

^Z^ *Paratrichodorus allius* nematodes were categorized as large (body length > 650 µm), medium (400–650 µm), and small (<400 µm) nematodes based on their body length. ^y^ The body length and the body width of the nematode were measured under the Zeiss Axio Scope A1 microscope with camera AxioCam 105 Color using the software, ZEISS ZEN 3.0 (blue edition) (Carl Zeiss Microscopy GmbH, Oberkochen, Germany) and the presented values were the range of length and width among ten individual nematodes. ^x^ Mean copy is the average DNA copies per µL of ten biological samples followed by standard deviation. The mean values with same letter were not statistically different (*p* > 0.05) in accordance with the least square means (LS means) adjusted for multiple comparisons using the Tukey—Kramer method.

**Table 2:** Soil pre-treatment effect on nematode DNA quantification by ddPCR.

Trt ^Z^	Pre-Trt wt (g) ^y^	After Trt wt (g) ^y^	Wt. Loss (%)	Mean Copy (DNA Copies/µL ± SD) ^X^
NT	-	-	-	275.42 ± 375.18 a
RT	25	24.06	3.76	122.99 ± 195.46 ab
OD	25	24.39	2.43	208.14 ± 208.10 ab
AC	25	24.13	3.48	1.89 ± 1.09 b

^Z^ Trt (treatments) included NT (no treatment, fresh soil), RT (air-dried at room temperature of 25 °C for 24 h), OD (oven-dried at 37 °C for 24 h), and AC (autoclaved at 121 °C for 20 min). In the study, naturally nematode-infested field soils were subjected to treatments and soil DNA was extracted using the DNeasy PowerSoil Pro Kit (Qiagen). ^y^ Pre-treatment weight and after treatment weight were averages of five soil samples. ^X^ Mean copy is the average DNA copies per µL of fifteen ddPCR reactions (five biological x three technical replicates) followed by standard deviation. The mean values followed by the same letter were not statistically different (*p* > 0.05) according to the least square means (LS means) adjusted for multiple comparisons using the Tukey–Kramer method.

## Data Availability

Data generated or analyzed during this study are included in this article.

## Supplementary Materials

The following supporting information can be downloaded at: https://www.mdpi.com/article/10.3390/ijms25063104/s1.
