# Supplementary material for: Assessment of Common Factors Associated with Droplet Digital PCR (ddPCR) Quantification of Paratrichodorus allius in Soil
Source: Int J Mol Sci. 2024 Mar 7;25(6):3104. doi: 10.3390/ijms25063104 (PMC10970607; doi:10.3390/ijms25063104)
Supplement: Supplementary file 1 [file ijms-25-03104-s001.zip › ijms-2876916-supplementary.pdf]

Supplementary materials

Supplementary Table S1: *Paratrichodorus allius* nematode population in soils determined by traditional extraction and microscopic counting methods.

Storage condition	Storage time	Nematode count (Mean ± SD)[z]
	0-month	43 ± 34 a
4⁰C	1-month	32 ± 28 ab
	2-months	22 ± 19 b
	3-months	19 ± 21 b

[z] Nematodes were extracted from 200 g of soil by sieving, sugar flotation and centrifugation methods followed by microscopic counts. The nematode count data in the table is the average number of nematodes from 15 samples (5 biological rep X 3 technical reps) followed by standard deviation. Same letters following the mean values are not significantly different at *P* <0.005.

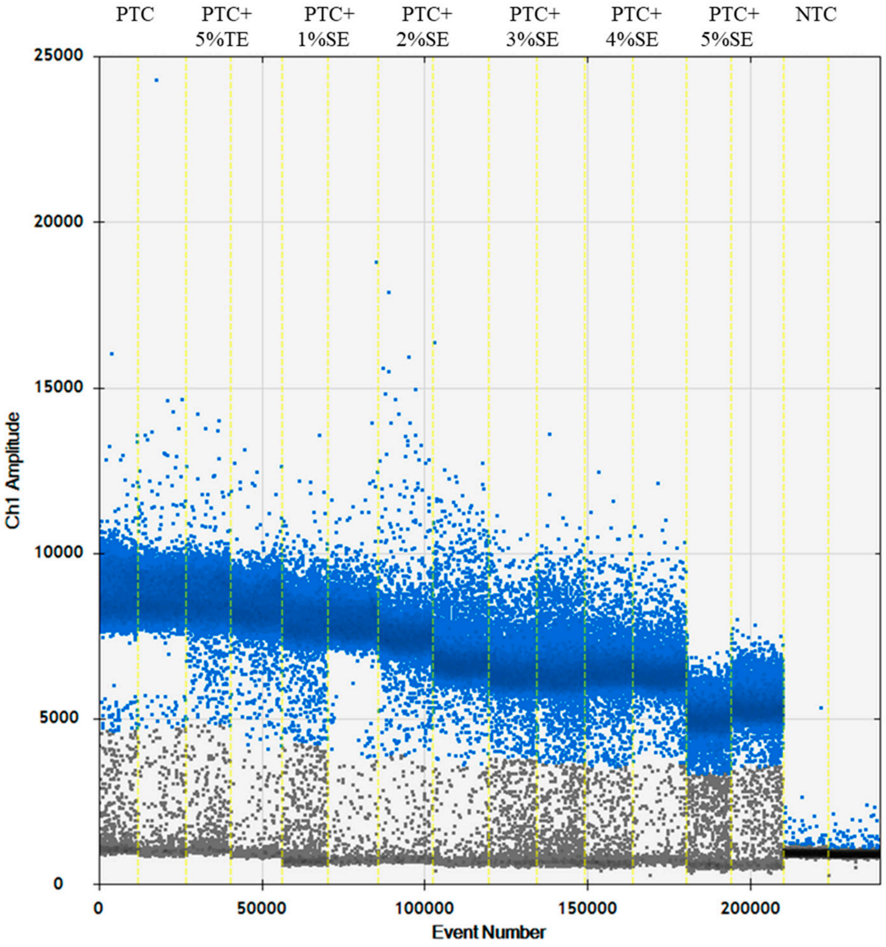

Supplementary Figure S1: Inhibitory effects of varying concentrations of soil extract on ddPCR. Positive template control (PTC) contained only the amplicon derivatives (obtained with the *Paratrichodorus allius* specific primers PaF11/PaR12, products confirmed by gel electrophoresis, and further validated by sequencing), without TE or SE. Soil extract (SE) was prepared from nematode-free field soil with 1X Tris EDTA (TE) buffer (pH 8.0) in a 1:1 (w/v) ratio. Varying concentrations of SE for ddPCR reactions were achieved by incorporating calculated volumes of stock SE to the ddPCR reaction mixture containing PTC. NTC was the no-template control in which nuclease-free double-

distilled water was added as template DNA. The graph presented the ddPCR derived amplitude plots of two biological samples run for each treatment. The trends of decreasing amplitudes with increasing concentrations of SE can be observed in the graph.

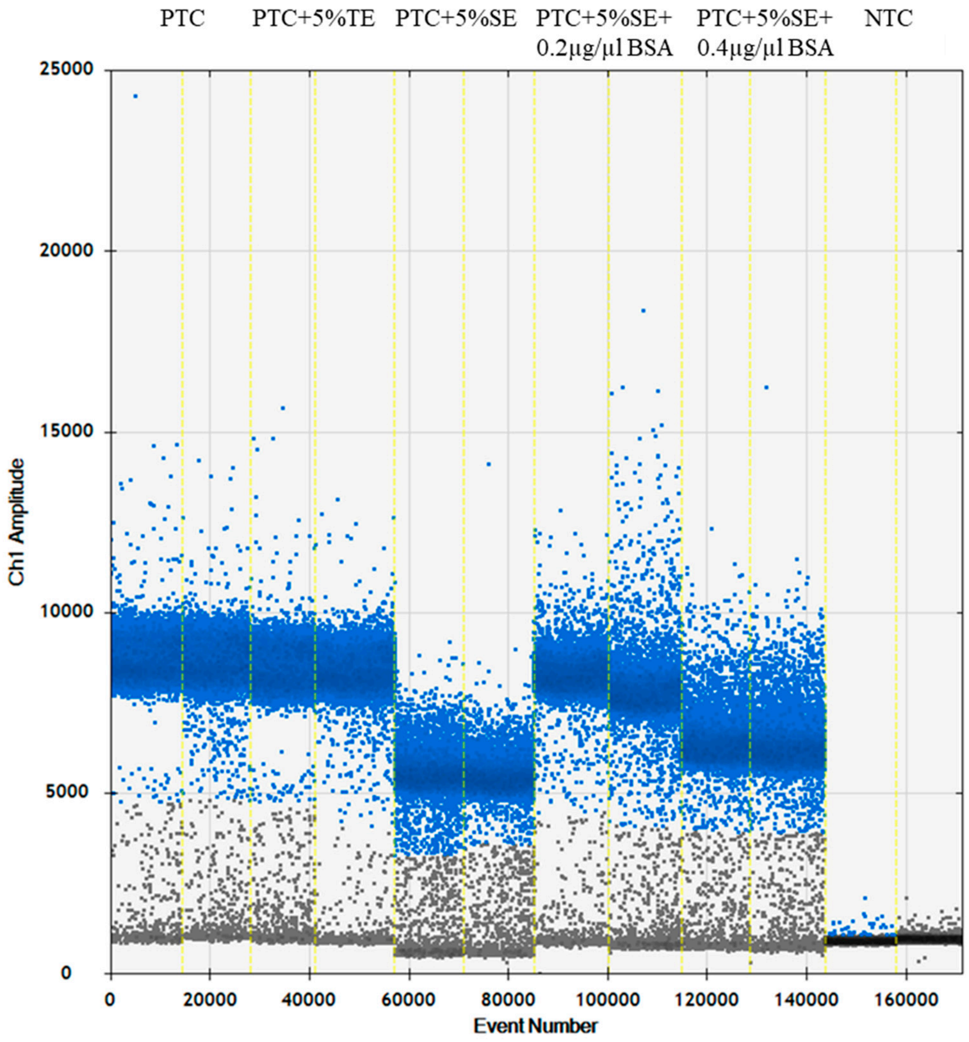

Supplementary Figure S2: Neutralizing effect of BSA on inhibition from soil extract on ddPCR. Positive template control (PTC) contained only the amplicon derivatives (obtained with the *Paratrichodorus allius* specific primers PaF11/PaR12, products confirmed by gel electrophoresis, and further validated by sequencing), but no other additives such as SE, TE or BSA. Soil extract (SE) was prepared from nematode-free field soil with 1X Tris EDTA (TE) buffer (pH 8.0) in a 1:1 (w/v) ratio. 5%SE with PTC was achieved by adding 1µl of stock SE to the 20µl reaction volume for ddPCR. Bovine serum albumin (BSA) concentrations of 0.2µg/µl and 0.4µg/µl in ddPCR were achieved by incorporating 0.4µl and 0.8µl of stock solution (10µg/µl) in 20µl ddPCR reaction volume, respectively. NTC was the no-template control in which nuclease-free double-distilled water was added as template DNA. The graph presented the ddPCR derived amplitude plots of two biological samples run for each treatment. The decreased amplitudes of the positive droplets (blue dots) with 5% SE and the reinstatement of the amplitudes comparable to PTC by BSA can be observed in the graph.
